# Effect of seat cushion resilience and hardness on lower-limb loading during sit-to-stand

**DOI:** 10.1186/s42490-026-00115-w

**Published:** 2026-06-17

**Authors:** Yusuke Onoda, Ami Ogawa

**Affiliations:** 1 School of Science for Open and Environmental Systems, Graduate School of Science and Technology, Keio University, Yokohama, 2238522 Japan; 2 Department of System Design Engineering, Keio University, 3-14-1 Hiyoshi, Kohoku-ku, Yokohama, 2238522 Japan

**Keywords:** Sit-to-stand, Resilience, Hardness, Seat cushion, Lower-limb loading

## Abstract

**Background:**

Seat cushion materials affect the mechanical demands of sit-to-stand (STS) movements; however, the effects of specific material properties, such as resilience and hardness, remain unclear. Understanding how these factors influence lower-limb joint moments and movement strategies during STS may contribute to the development of seat designs that assist individuals with reduced lower-limb strength. Therefore, this study aimed to clarify the fundamental mechanical effects of seat cushion resilience and hardness on STS.

**Methods:**

Fifteen healthy young adults performed STS from five polyurethane foam cushions that differed in resilience (14–55%) and 40% compression hardness (66–336 N). The material ranges were determined with reference to technical documents and a published patent specification to ensure they were within the range commonly used in everyday seating products. Kinematic and kinetic data were collected using a motion capture system and two force plates. Net joint moments were calculated via inverse dynamics, and differences among seat conditions were analyzed using repeated-measures ANOVA or the Friedman test, with Bonferroni-adjusted pairwise comparisons (α = 0.05).

**Results:**

Seat resilience significantly affected the peak hip and knee extensor moments (*p* < 0.01, η² = 0.35–0.48). High-resilience cushions delayed seat-off timing and maintained greater seat reaction force at the timings of peak hip and knee extensor moments. In contrast, seat hardness mainly influenced horizontal center-of-mass (COM) velocity and hip joint moment (*p* < 0.01, η² = 0.31–0.37), with softer seats producing larger values.

**Conclusion:**

High-resilience cushions delayed seat-off and maintained buttock support for a longer duration, thereby reducing the peak hip and knee extensor moments. In contrast, softer seats promoted a strategy involving greater horizontal momentum generation by the upper body, which consequently required an increased hip extensor moment to decelerate this momentum. Cushions with a resilience of ≥ 53% and 40% compression hardness of ≥ 180 N effectively reduced lower-limb joint loading. The results of this study provide fundamental insights that may contribute to future research on chair design and cushion selection in clinical and caregiving environments.

**Clinical trial:**

Not applicable.

**Supplementary Information:**

The online version contains supplementary material available at 10.1186/s42490-026-00115-w.

## Introduction

The sit-to-stand (STS) movement is one of the most frequently performed actions in daily life. To complete STS, the center of mass (COM) should be moved horizontally from the broad base of support formed by the buttocks and feet to the narrow base of support formed solely by the feet, while also moving vertically [1]. Thus, sufficient lower-limb strength is required to perform STS [2, 3]. However, as lower-limb strength generally declines after the age of 50 [4], STS can be a demanding movement for older adults [5]. Moreover, knee osteoarthritis, which is estimated to affect 18% of women and 10% of men over the age of 60 [6], is associated with decreased quadriceps muscle strength [7], making STS more difficult. As STS contributes to expanding the range of activities, including walking, the ability to perform STS is crucial for functional independence [8].

Accordingly, to assist individuals with reduced lower-limb strength, STS has been examined from both strategic perspectives, focusing on adjustments such as foot placement [9–11] and trunk movement [12–14], and environmental perspectives, focusing on chair design factors such as seat height [15–19] and the presence of armrests [16, 20, 21].

Yoshioka et al. generated and analyzed more than 5,000,000 STS movements performed from a chair with a height of 0.4 m without using any arm support or countermovement [22]. Their results suggested that, when foot placement, arm use, and chair height were consistent, the combined peak hip and knee extensor moments must consistently exceed approximately 1.53 Nm/kg across various movement patterns. Furthermore, they reported that the relative contribution of the hip and knee extensor moments changes with the degree of trunk flexion—greater trunk flexion increases the hip joint moment while decreasing the knee joint moment. Based on these findings, in order to simultaneously reduce the mechanical load on both joints, it is important to optimize the seating environment, rather than relying on compensatory strategies such as excessive forward trunk flexion. Previous studies on STS and seating have primarily examined the effects of seat height [15–19], seat angle [15, 23, 24], and armrests [16, 20, 21]. Rodosky et al. investigated STS from seat heights corresponding to 65%, 80%, 100%, and 115% of knee joint height, and demonstrated that the maximum knee joint moment from the lowest seat height was nearly twice that from the highest seat height [18]. Shin et al. demonstrated that increasing the seat inclination angle led to earlier activation of muscles such as the rectus femoris (RF) and tibialis anterior (TA), suggesting that this adjustment facilitates smoother execution of STS [24]. Alexander et al. compared older adults with and without difficulty standing up and found that those with difficulty exerted significantly greater handrail support forces relative to body weight (0.29 vs. 0.19), suggesting that they compensated for insufficient lower-limb strength by relying more on their upper limbs [20]. In addition to modifications of chair design parameters, assistive devices mounted on the seat to facilitate STS have also been investigated. Wretenberg et al. analyzed STS in patients with knee osteoarthritis using a spring-loaded flap seat, and reported that the peak knee and hip joint moments decreased from 52.7 to 33.0 Nm and from 50.3 to 34.9 Nm, respectively [25].

Several studies have examined the effects of seat cushions on STS. Sato et al. compared STS from a seat with a low-resilience urethane cushion (80 mm thick) and from a seat without a cushion, and found that the duration between the peak center of pressure at the rearfoot and that at the forefoot was significantly longer when using the cushioned seat [26]. That is, they suggested that using a cushion during STS prolongs the duration from seat-off to postural stabilization. Anan et al. compared STS using cushions with thicknesses of 0, 30, 60, and 90 mm, and reported that as cushion thickness increased, trunk forward inclination became larger, while the load on the knee extensors simultaneously increased [27]. The effects of seat cushions on STS performance may be significant and, like seat height, should be considered an important factor in seating design. However, the mechanical effects of seat material properties on STS have not been fully clarified. Sato et al. compared a low-resilience cushion with a firm seat surface without cushion; however, the effects of seats with medium or high resilience on STS remain unclear [26]. To elucidate the mechanical influence of cushion resilience, it is essential to experimentally compare materials with different levels of resilience. Anan et al. pointed out that increased buttock immersion restricts pelvic motion and consequently increases movement inefficiency [27]. Considering that pelvic immersion is affected not only by cushion thickness but also by hardness, cushion hardness may also influence STS performance. Therefore, in the present study, we focused on the mechanical effects of cushion resilience and hardness on STS performance under standardized seating conditions.

Soft polyurethane foam is a typical material with excellent cushioning properties and a wide variety of mechanical properties. Owing to its low weight and high mechanical strength, urethane is used in many applications in the modern furniture industry, including mattresses, sofas, and seat cushions [28]. According to ISO standards, the primary physical properties of urethane include density (ISO 845), tensile strength (ISO 1798), hardness (ISO 2439), air permeability (ISO 4638), and resilience (ISO 8307). In addition, there are other properties used to evaluate durability and long-term performance. Among these physical properties, we focused on resilience and hardness as factors likely to influence STS performance, and formulated two hypotheses accordingly.

Firstly, we hypothesized that a high-resilience seat material would reduce the peak hip and knee extensor joint moments during STS. The spring-loaded flap seat, which propels the body upward and forward through elastic force, reduced the hip and knee joint moments to approximately 60–70% of those during normal STS [25]. Although such a mechanism differs from material resilience itself, it suggests that elastic energy returned from the seat surface can help reduce joint loading. Therefore, even for a conventional seat without springs, we expected that a high-resilience cushion would generate greater upward reaction force on the body, facilitate smoother COM movement, and consequently reduce the mechanical loads on the hip and knee joints.

Secondly, we hypothesized that when standing up from a softer seat surface, a strategy involving greater trunk flexion angle and higher horizontal velocity of the COM would be preferred during STS. Increased buttock immersion restricts pelvic motion during STS, thereby necessitating greater use of the upper body to generate momentum and resulting in increased forward trunk inclination [27]. Thus, softer seats may induce greater forward trunk flexion accompanied by faster forward momentum of the trunk as a compensatory strategy.

This study aimed to clarify the fundamental mechanical effects of seat cushion resilience and hardness on STS by recruiting young adults instead of elderly individuals, who exhibit greater variability in physical and cognitive functions. The findings can serve as baseline data for future comparative studies involving older adults or clinical populations.

## Methods

### Subjects

Fifteen healthy young adults (11 males, 4 females, age 23 ± 1 years, body height 1.72 ± 0.07 m, body mass 60.0 ± 7.7 kg) participated in this study. The sample size was determined with reference to previous studies that compared STS under different seat or environmental conditions. Most of these studies included fewer than 15 participants [13, 17, 26, 27, 29]. All participants were fully informed of the purpose and methods of the study, and written informed consent was obtained from each participant before measurements were taken. This study was approved by the Ethical Review Committee of the Faculty of Science and Technology at Keio University (Approval No. 2023-048) on July 7, 2023.

### Measurements

#### Seat material

Five types of polyurethane foam cushions were selected from commercially available materials to provide a well-distributed range of resilience and hardness representative of flexible polyurethane foams used for seating applications: MMT48, 35 S, and 35 H, manufactured by Toyo Quality One Corporation (Saitama, Japan); EMM and EFF, manufactured by Inoac Corporation (Tokyo, Japan). The planar dimensions of the cushions (300 × 400 mm) were matched to the force plate (TF-3040, Tec Gihan Co., Ltd., Japan). The thickness was set to 80 mm — a practical value that prevents bottoming out and falls within the range commonly used in everyday seating products.

The resilience was measured according to a procedure based on ISO 8307. Three specimens (100 mm × 100 mm × 80 mm) of the same cushion type were prepared. As a preconditioning process, each specimen was compressed twice consecutively at a speed of 0.4–6 mm/s until its thickness reached 20–25% of the original value, and then allowed to rest for 10 ± 5 min. Subsequently, a steel ball (diameter: 16 mm, mass: 16.8 g) was dropped without rotation from a height of 500 mm onto the center of each specimen. The rebound height of the ball was measured, and the rebound ratio was calculated as the ratio of the rebound height to the initial drop height (500 mm). For each specimen, three measurements were performed within one minute, and the median value was calculated. Finally, the overall median of the three medians obtained from the three specimens was defined as the resilience.

The hardness was measured according to a procedure based on ISO 2439. The urethane cushion specimen (380 mm × 380 mm × 80 mm) was compressed to 30% of its original thickness (strain of 70%) at a speed of 100 ± 20 mm/min. The compression plate was then returned to its initial position at the same speed. This compression and recovery procedure was repeated twice more as preconditioning cycles. Immediately after the completion of the preconditioning cycles, the specimen was compressed to 60% of its original thickness (strain of 40%) at a speed of 100 ± 20 mm/min. The compression was maintained for 30 ± 1 s, after which the force at that moment was recorded and defined as the hardness.

**Fig. 1:**
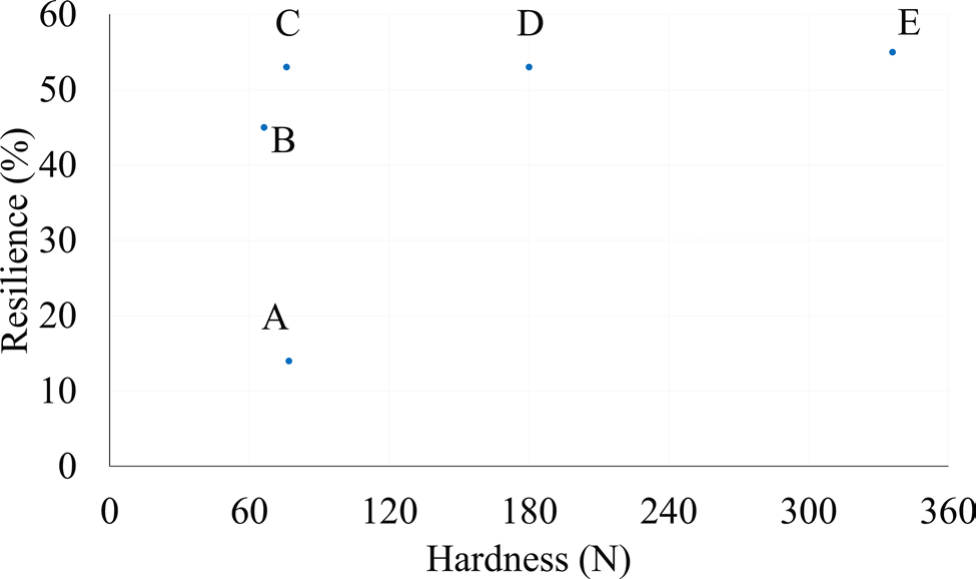
Resilience and hardness of cushions. Comparison of the physical properties of five urethane cushions (seats A–E). Seats A–C have approximately the same hardness, and the effects of resilience can be compared. Seats C–E exhibited approximately the same resilience, and the effects of hardness were compared. (Resilience [%], Hardness [N]) = A(14, 77), B(45, 66), C(53, 76), D(53, 180), E(55, 336)

Prior to the main experiment, the resilience and hardness of each cushion were measured by the BOKEN Quality Evaluation Institute. MMT48, EFF, 35S, 35H, and EMM were labeled as seats A, B, C, D, and E, respectively (Fig. 1). According to technical documents and a published patent specification (Japan Patent Application JP2023130692A, 2023), flexible polyurethane foams typically exhibit 25% indentation hardness of 10–600 N and rebound resilience of 5–80% [30]. Although these reference values are based on 25% indentation testing, the hardness values measured at 40% indentation in the present study (66–336 N) and the resilience (14–55%) both fall within the expected range for soft to firm and low- to high-resilience seating foams.

#### Measurement sensors

An optical motion capture system (VICON, Vicon Motion Systems, Oxford, UK), acquired joint position information during STS. Thirty-nine markers were attached to the subject according to the Vicon Full-Body Plug-In Gait Model, and ten cameras (eight Vicon Bonita B10 and two Vicon Vero) tracked all the marker position coordinates. Simultaneously, reaction forces at the buttocks and feet were measured using the two force plates. VICON and the force plates were synchronized with the frame rate of VICON set to 100 fps and that of the force plates set to 1000 fps. All raw data obtained from VICON and the force plates were smoothed using a fourth-order zero-lag Butterworth low-pass digital filter. The cutoff frequencies were determined by residual analysis and set to 8 Hz for the VICON coordinate data and 25 Hz for the ground reaction force (GRF) data [31].

#### Measurement conditions

**Fig. 2:**
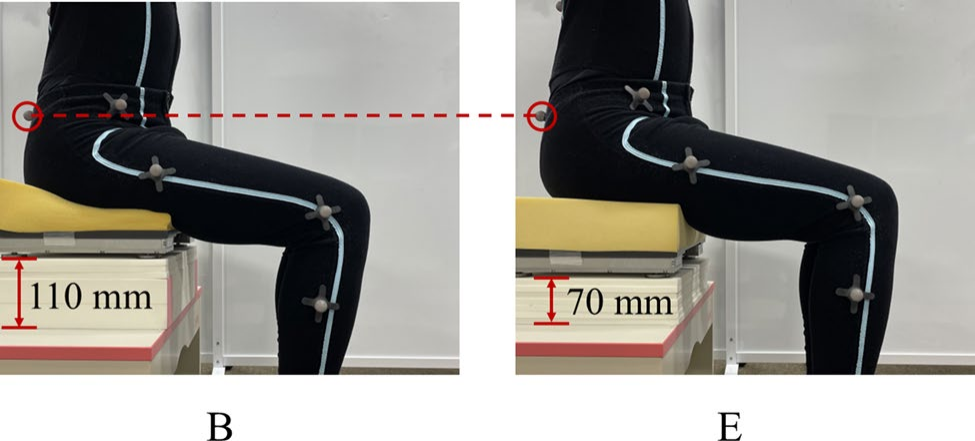
Seats after height adjustment. The height of the right posterior superior iliac spine marker (RPSI) was kept constant across conditions. A flat rehabilitation chair (height: 300 mm) was used to ensure stability of the cushions and the force plate. Polyethylene foam was inserted between the force plate and the chair to standardize the distance from the floor to the buttocks

**Fig. 3:**
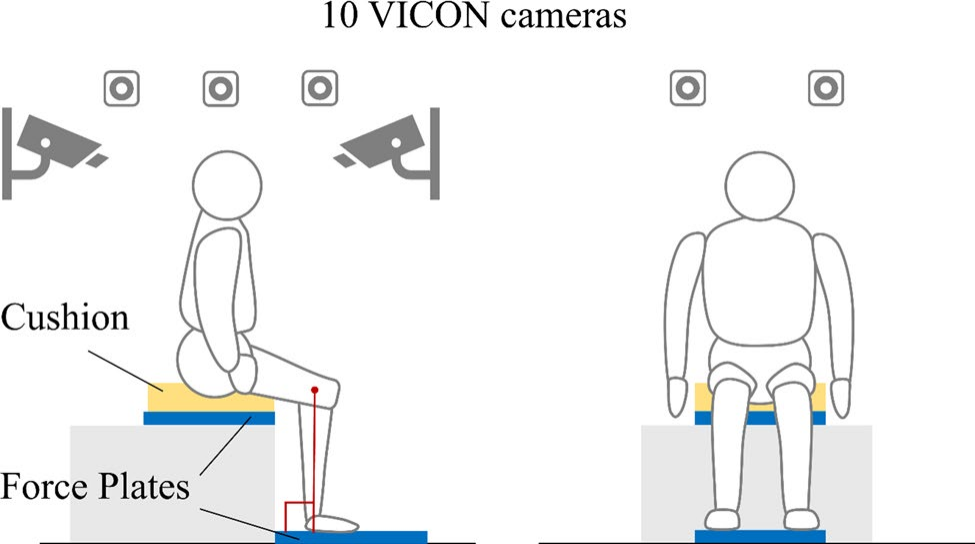
Placement of the experimental apparatus and sitting posture of the subject. Subject seated with the feet on a force plate and the buttocks on a cushion above the other force plate. Ten VICON cameras placed above and surrounding the seated subject

Because the force applied to the handrail varies [20] and the use of the hands affects the magnitude of lower-limb joint moments [16], the use of handrails and arm countermovement was not permitted in this study. In previous studies, participants were instructed to cross their arms while standing up [26, 27]; however, in this study, to prevent the sternum marker (STRN) from being obscured by the arms, participants were instructed to keep their arms alongside the trunk throughout the movement. Cushion sinking must be considered because a lower seat height increases the mechanical demand required to stand up [15–19]. In this study, to standardize the distance from the floor to the buttocks under each seat condition, the height of the right posterior superior iliac spine marker (RPSI) was monitored in real time using Vicon Nexus v2.15 (Vicon Motion Systems, Oxford, UK), and the seat height was adjusted accordingly (Fig. 2). Although previous studies defined the standard height as that corresponding to a knee flexion angle of 90° on the harder seat [26], under the conditions of the present experiment, this made it difficult to stand up from the low-resilience seat. Therefore, the standard RPSI height was set as the height when sitting on the hardest seat (seat E) with a knee flexion angle of 80°. In addition, the participants sat with their shank perpendicular to the floor [26, 27]. A digital goniometer was used to measure these reference angles. Footprints were marked on the force plate so that the feet were positioned shoulder-width apart with the toes pointing outward at approximately 10°, ensuring consistent anterior–posterior foot placement across trials (Fig. 3). Each participant performed sufficient practice followed by two main trials for each seat condition. Rest periods were provided when either the participant or the supervising instructor judged them to be necessary. The measurement order of the seat conditions was randomized across participants.

#### Evaluation indicators

**Fig. 4:**
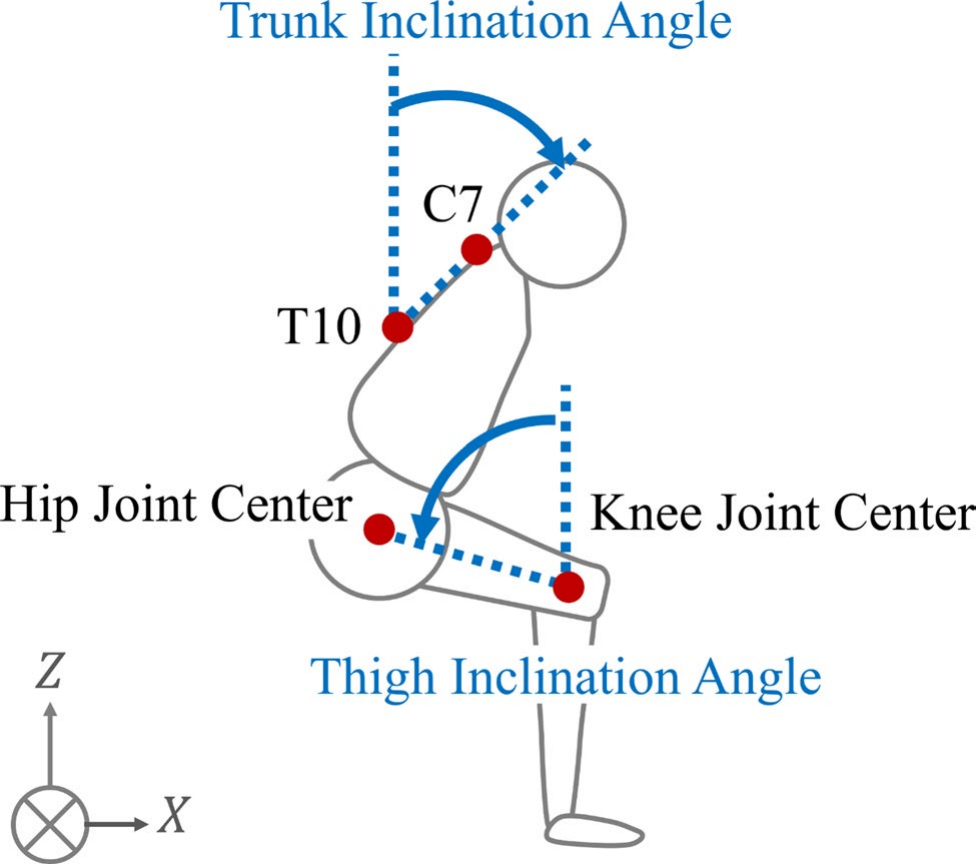
Definition of trunk and thigh inclination angles. The trunk inclination angle was defined as the angle between the vector connecting the C7 and T10 markers and the global vertical axis (Z-axis). The thigh inclination angle was defined as the angle between the vector connecting the hip and knee joint centers and the global vertical axis. Marker and joint center positions were determined based on the Vicon Plug-in Gait model. Both angles were calculated in the sagittal plane of the global coordinate system (X: anterior–posterior, Y: medial–lateral, Z: vertical)

To evaluate STS under each seat condition, several kinematic and kinetic parameters were analyzed. To verify the two hypotheses stated in the introduction, the peak values of trunk inclination angle, COM velocities (horizontal and vertical components), and lower-limb joint moments (ankle plantar flexor, knee extensor, and hip extensor) were calculated. In the global coordinate system of Vicon (X: anterior–posterior, Y: medial–lateral, Z: vertical), the trunk inclination angle was defined as the angle between the vector connecting the C7 and T10 markers and the global vertical axis (Z-axis) (Fig. 4). In addition, the vertical GRF was analyzed as a kinetic indicator representing overall lower-limb exertion. The peak vertical GRF was expected to increase when participants generated greater lower-limb muscular force [32] or when a greater portion of the upper-body momentum was transferred to the lower limbs. The peak lower-limb joint moments and vertical GRF were normalized by dividing them by the subject’s body weight.

To identify the factors contributing to these peak values, additional analyses were conducted. First, to elucidate how seat surfaces with different material properties support the body weight during STS, the temporal changes in seat reaction force (SRF) and GRF were examined. In this study, three key time points— the onset of the movement, the time of peak hip moment (PHM), and the time of peak knee moment (PKM)—were defined, and the SRF and GRF values at three points were calculated. Furthermore, to compare the movement mechanisms of the thigh segment in contact with the seat surface, the thigh inclination angle was calculated at the three key time points. The thigh inclination angle was defined as the angle between the vector connecting the hip and knee joint centers and the global vertical axis (Z-axis) (Fig. 4). In addition, the timings of seat-off, PHM, and PKM under each seat condition were calculated as percentages of the total STS duration to examine whether seat material properties affected the temporal coordination of lower-limb joint moments during STS. Following the method proposed by Gross et al., the onset and termination of the STS movement were defined as the moments when the trunk angular velocity first exceeded 5.7°/s in the forward or backward direction, respectively. Seat-off was defined as the instant when the SRF fell below 3 N.

Marker, Joint, and COM positions were obtained using Vicon Nexus v2.15 (Vicon Motion Systems, Oxford, UK), and all calculations were performed in MATLAB R2023a (MathWorks Inc., Natick, MA, USA). The method used to calculate joint moments is detailed in the Appendix.

### Statistical analysis

The mean values obtained from two main trials were used for statistical analysis. As participants performed sufficient practice before the main trials under each condition, the variability between the two trials was expected to be minimal.

To examine the effect of the resilience of the seat on STS, significance tests were conducted for seats A–C, which had approximately the same hardness but different resilience. In addition, to examine the effect of seat hardness on STS, tests of significant differences were conducted for seats C–E, which had approximately the same resilience but different hardness values. First, the Shapiro–Wilk test was conducted to examine the normality of each evaluation indicator for each seat condition. The statistical methods were selected based on whether the data followed a normal distribution. If the normality assumption was satisfied, a repeated-measures ANOVA was performed to assess differences among seat conditions. When the assumption of sphericity was violated, the Greenhouse–Geisser correction was applied. Post hoc comparisons were conducted using the Bonferroni correction. Otherwise, the Friedman test was performed, and Bonferroni-adjusted pairwise comparisons were applied. A statistical significance level was set at *p* < 0.05. IBM SPSS Statistics v.29 software (IBM, Armonk, NY, USA) was used for all statistical analyses.

## Results

Statistical outcomes, including p-values, effect sizes, and significant seat-pair differences for all indicators, are summarized in Table 1. The detailed results for each factor, seat resilience and seat hardness, are described in the following subsections.

**Table 1:** Statistical summary of seat condition effects on STS indicators

Indicators	Seat Group	Statistical Test	*p*-value	Effect Size	Significant Pairs
Peak horizontal COM velocity	C-E	Repeated-measures ANOVA	0.002	$\:{\eta\:}^{2}$= 0.37	C > D (*p* = 0.002) C > E (*p* = 0.022)
Peak vertical GRF	A-C	Repeated-measures ANOVA	0.005	$\:{\eta\:}^{2}$= 0.32	A > C (*p* = 0.004)
	C-E	Friedman test	0.004	Kendall’s W = 0.37	C > D (*p* = 0.003)
Peak hip moment	A-C	Repeated-measures ANOVA	0.003	$\:{\eta\:}^{2}$= 0.35	A > B (*p* = 0.026) A > C (*p* = 0.003)
	C-E	Repeated-measures ANOVA	0.005	$\:{\eta\:}^{2}$= 0.31	C > D (*p* = 0.025) C > E (*p* = 0.009)
Peak knee moment	A-C	Repeated-measures ANOVA	< 0.001	$\:{\eta\:}^{2}$= 0.48	A > B (*p* = 0.023) A > C (*p <* 0.001)
SRF at PHM	A-C	Friedman test	< 0.001	Kendall’s W = 0.88	A < B (*p* = 0.006) A < C (*p <* 0.001)
	C-E	Friedman test	< 0.001	Kendall’s W = 0.67	C > E (*p* = 0.002) D > E (*p <* 0.001)
SRF at PKM	A-C	Friedman test	< 0.001	Kendall’s W = 0.94	A < B (*p* = 0.010) A < C (*p <* 0.001)
	C-E	Friedman test	< 0.001	Kendall’s W = 0.48	C > E (*p* = 0.003) D > E (*p =* 0.003)
GRF at PHM	C-E	Friedman test	0.006	Kendall’s W = 0.34	C > D (*p* = 0.010) C > E (*p* = 0.032)
GRF at PKM	C-E	Friedman test	0.008	Kendall’s W = 0.32	C > D (*p* = 0.006)
Thigh Inclination Angle at the Onset	C-E	Repeated-measures ANOVA	0.002	$\:{\eta\:}^{2}$= 0.35	C > E (*p* = 0.002)
Thigh Inclination Angle at PHM	A-C	Repeated-measures ANOVA	0.003	$\:{\eta\:}^{2}$= 0.35	A > C (*p* = 0.002)
	C-E	Repeated-measures ANOVA	< 0.001	$\:{\eta\:}^{2}$= 0.55	C > D (*p <* 0.001) C > E (*p <* 0.001)
Thigh Inclination Angle at PKM	C-E	Repeated-measures ANOVA	0.001	$\:{\eta\:}^{2}$= 0.38	C > D (*p* = 0.010) C > E (*p =* 0.002)
The timing of seat-off	A-C	Repeated-measures ANOVA	< 0.001	$\:{\eta\:}^{2}$= 0.51	A < B (*p <* 0.001) A < C (*p <* 0.001)
The timing of PKM	C-E	Friedman test	0.031	Kendall’s W = 0.23	C < E (*p =* 0.032)

COM (center-of-mass), GRF (ground reaction force), SRF (seat reaction force), PHM (peak hip moment), PKM (peak knee moment)

(Resilience [%], Hardness [N]) = A(14, 77), B(45, 66), C(53, 76), D(53, 180), E(55, 336)

A–C: comparison of seat resilience; C–E: comparison of seat hardness

### Effect of seat resilience on STS mechanics

Seat resilience significantly affected the peak hip and knee joint moments as well as the peak vertical GRF (Table 2). The peak hip joint moment was significantly greater for seat A than for seats B (*p* = 0.026) and C (*p =* 0.003). Likewise, the peak knee joint moment was significantly greater for seat A than for seats B (*p* = 0.023) and C (*p* < 0.001). Furthermore, the peak vertical GRF was significantly higher for seat A than for seat C (*p* = 0.004).

The vertical SRF also differed significantly among seats A–C at both PHM and PKM (Table 3). For seat C, the vertical SRF declined from 8.75 ± 0.38 N∙kg^-1^ at the onset of STS to 0.72 ± 0.18 N∙kg^-1^ at PHM (≈ 8% of the initial value) and 0.47 ± 0.16 N∙kg^-1^ at PKM (≈ 5%). In contrast, for seat A, the corresponding values were 8.65 ± 0.47 N∙kg^-1^, 0.21 ± 0.24 N∙kg^-1^ (≈ 2%), and 0.01 ± 0.01 N∙kg^-1^, indicating that, by the time of PKM, seat support was virtually eliminated. Accordingly, the vertical SRF at both PHM and PKM was significantly higher for seat C than for seat A (*p* < 0.001). A comparable trend was also evident between seats A and B, whereby higher seat resilience was associated with larger SRF at both PHM and PKM.

**Table 2:** Comparison of kinetic parameters during STS

Seats	Peak Vertical GRF (*N*∙kg^− 1^)		Peak Joint Moments (*N*∙m∙kg^− 1^)					
			Hip Extensor		Knee Extensor		Ankle Plantar Flexor	
	Mean ± SD	(95% CI)	Mean ± SD	(95% CI)	Mean ± SD	(95% CI)	Mean ± SD	(95% CI)
A	12.91 ± 0.80^c^	(12.47, 13.35)	1.00 ± 0.13^b, c^	(0.93, 1.07)	0.79 ± 0.13^b, c^	(0.72, 0.86)	0.29 ± 0.11	(0.22, 0.35)
B	12.66 ± 0.89	(12.17, 13.16)	0.95 ± 0.13^a^	(0.88, 1.02)	0.74 ± 0.13^a^	(0.67, 0.82)	0.28 ± 0.11	(0.22, 0.34)
C	12.51 ± 0.81^a, d^	(12.06, 12.95)	0.93 ± 0.14^a, d,e^	(0.85, 1.01)	0.71 ± 0.13^a^	(0.64, 0.78)	0.26 ± 0.13	(0.19, 0.33)
D	11.97 ± 0.70^c^	(11.58, 12.35)	0.88 ± 0.11^c^	(0.81, 0.94)	0.68 ± 0.13	(0.60, 0.75)	0.27 ± 0.14	(0.19, 0.34)
E	12.16 ± 0.82	(11.70, 12.62)	0.87 ± 0.13^c^	(0.80, 0.94)	0.70 ± 0.14	(0.63, 0.78)	0.28 ± 0.10	(0.22, 0.33)

GRF (ground reaction force)

(Resilience [%], Hardness [N]) = A(14, 77), B(45, 66), C(53, 76), D(53, 180), E(55, 336)

Superscript letters (a–e) indicate significant differences between seats (p < 0.05). See Table 4 for details

**Table 3:** Comparison of SRF (seat reaction force) among the three key time points

Seats	SRF at the Onset (*N*∙kg^− 1^)		SRF at PHM (*N*∙kg^− 1^)		SRF at PKM (*N*∙kg^− 1^)	
	Mean ± SD	(95% CI)	Mean ± SD	(95% CI)	Mean ± SD	(95% CI)
A	8.65 ± 0.47	(8.39, 8.91)	0.21 ± 0.24^b, c^	(0.08, 0.34)	0.01 ± 0.01^b, c^	(0.004, 0.15)
B	8.62 ± 0.36	(8.42, 8.82)	0.58 ± 0.17^a^	(0.48, 0.67)	0.30 ± 0.13^a^	(0.22, 0.37)
C	8.75 ± 0.38	(8.53, 8.96)	0.72 ± 0.18^a, e^	(0.62, 0.82)	0.47 ± 0.16^a, e^	(0.38, 0.56)
D	8.73 ± 0.44	(8.49, 8.98)	0.82 ± 0.38^e^	(0.62, 1.03)	0.50 ± 0.36^e^	(0.30, 0.70)
E	8.78 ± 0.40	(8.55, 9.00)	0.32 ± 0.31^c, d^	(0.15, 0.50)	0.06 ± 0.11^c, d^	(0.002, 0.13)

PHM (peak hip moment), PKM (peak knee moment)

(Resilience [%], Hardness [N]) = A(14, 77), B(45, 66), C(53, 76), D(53, 180), E(55, 336)

Superscript letters (a–e) indicate significant differences between seats (p < 0.05). See Table 4 for details

**Fig. 5:**
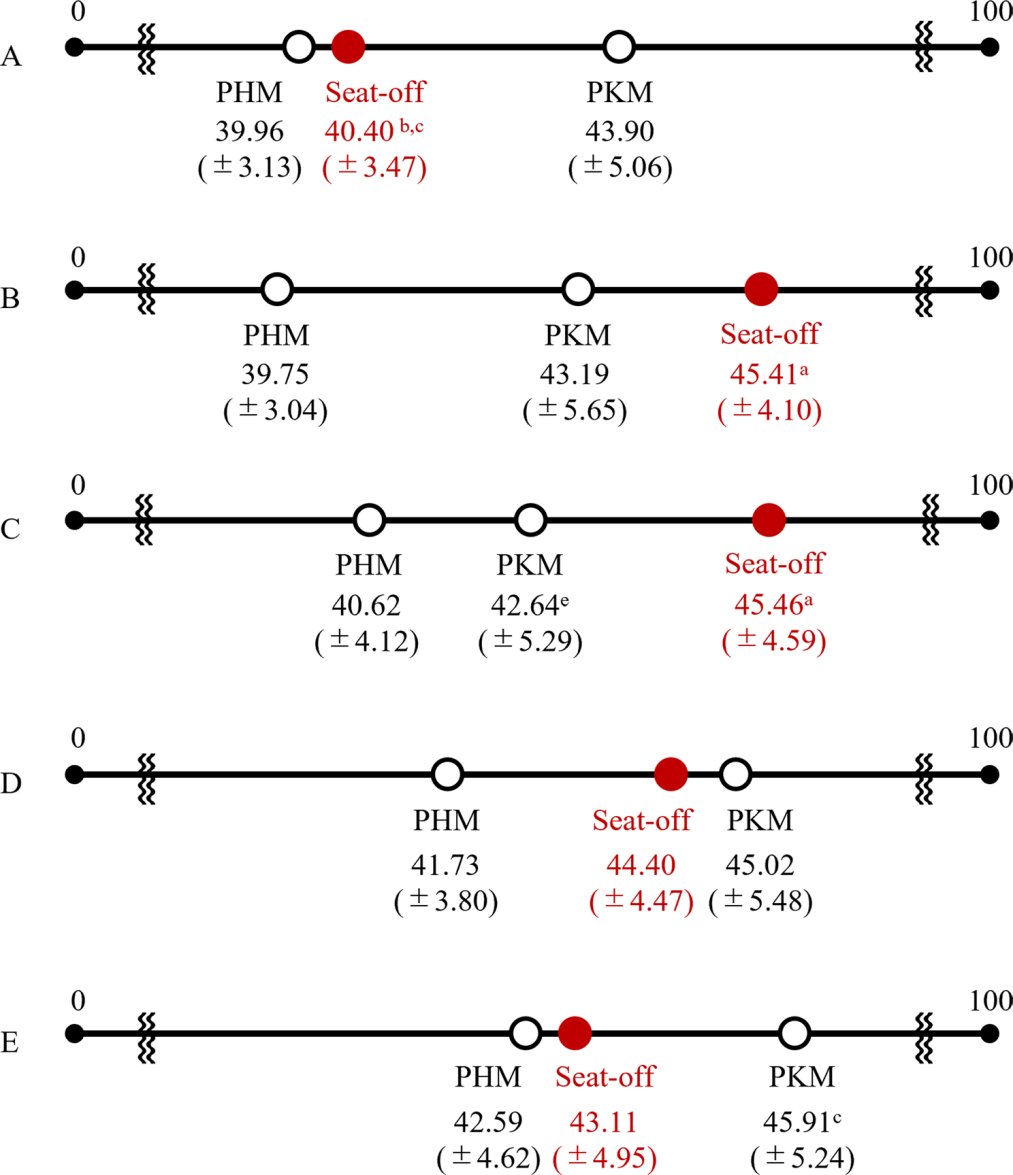
Temporal distribution of seat-off, PHM and PKM across seat conditions. The timing of seat-off, peak hip moment (PHM), and peak knee moment (PKM) is expressed as a percentage of the total STS duration for each seat condition

Seat resilience had a significant effect on the timing of seat-off (Fig. 5). The timing of seat-off was significantly earlier for seat A than for seats B (*p* < 0.001) and C (*p* < 0.001). Notably, seat-off occurred before PKM for seat A, whereas for seats B and C, seat-off was delayed until after PKM.

### Effect of seat hardness on STS mechanics

Seat hardness significantly affected the peak horizontal COM velocity (Table 4), the peak hip joint moment, and the peak vertical GRF (Table 2). The peak horizontal COM velocity was significantly higher for seat C than for seats D (*p =* 0.002) and E (*p =* 0.022). Similarly, the peak hip joint moment was significantly greater for seat C than for seats D (*p =* 0.025) and E (*p =* 0.009). The peak vertical GRF was likewise higher for seat C than for seat D (*p =* 0.003). The peak trunk inclination angle increased with decreasing seat hardness; however, the difference among seat conditions was not statistically significant (*p* = 0.127).

**Table 4:** Physical properties of each seat and comparison of kinematic parameters during STS

Seats	Resilience (%)	Hardness (*N*)	Peak Trunk Inclination Angle (°)		Peak COM Velocities (mm/s)			
					Horizontal		Vertical	
			Mean ± SD	(95% CI)	Mean ± SD	(95% CI)	Mean ± SD	(95% CI)
A	14	77	65.31 ± 12.65	(58.30, 72.32)	594.73 ± 83.14	(548.69, 640.77)	605.91 ± 130.52	(533.63, 678.19)
B	45	66	64.99 ± 11.28	(58.75, 71.24)	596.96 ± 92.05	(545.98, 647.94)	601.48 ± 125.49	(531.99, 670.97)
C	53	76	63.41 ± 11.99	(56.77, 70.05)	588.91 ± 79.36^d, e^	(544.96, 632.86)	577.17 ± 116.06	(512.90, 641.44)
D	53	180	62.85 ± 11.61	(56.42, 69.28)	543.11 ± 84.24^c^	(496.46, 589.75)	565.62 ± 126.10	(495.79, 635.45)
E	55	336	61.02 ± 9.17	(55.95, 66.10)	554.75 ± 80.17^c^	(510.35, 599.14)	596.91 ± 120.01	(530.45, 663.37)

COM (center-of-mass)

Statistical analyses were conducted among seats A–C and seats C-E, revealing significant differences in several parameters

^a^ Significantly different than seat A (*p* < 0.05)

^b^ Significantly different than seat B (*p* < 0.05)

^c^ Significantly different than seat C (*p* < 0.05)

^d^ Significantly different than seat D (*p* < 0.05)

^e^ Significantly different than seat E (*p* < 0.05)

The vertical GRF also differed significantly among seats C–E at both PHM and PKM (Table 5). At PHM, the GRF was significantly higher for seat C than for seats D (*p* = 0.010) and E (*p* = 0.032). Similarly, at PKM, seat C exhibited a significantly higher GRF than seat D (*p* = 0.006).

**Table 5:** Comparison of GRF (ground reaction force) among the three key time points

Seats	GRF at the Onset (*N*∙kg^− 1^)		GRF at PHM (*N*∙kg^− 1^)		GRF at PKM (*N*∙kg^− 1^)	
	Mean ± SD	(95% CI)	Mean ± SD	(95% CI)	Mean ± SD	(95% CI)
A	1.48 ± 0.41	(1.25, 1.71)	12.62 ± 0.91	(12.12, 13.12)	12.65 ± 0.80	(12.20, 13.09)
B	1.51 ± 0.31	(1.34, 1.68)	12.44 ± 0.92	(11.93, 12.95)	12.53 ± 0.96	(12.00, 13.06)
C	1.40 ± 0.35	(1.20, 1.59)	12.34 ± 0.80^d, e^	(11.90, 12.78)	12.41 ± 0.83^d^	(11.96, 12.87)
D	1.42 ± 0.37	(1.21, 1.62)	11.84 ± 0.67^c^	(11.47, 12.22)	11.71 ± 0.80^c^	(11.27, 12.16)
E	1.36 ± 0.34	(1.17, 1.55)	11.89 ± 0.93^c^	(11.38, 12.41)	12.03 ± 0.82	(11.57, 12.48)

PHM (peak hip moment), PKM (peak knee moment)

(Resilience [%], Hardness [N]) = A(14, 77), B(45, 66), C(53, 76), D(53, 180), E(55, 336)

Superscript letters (a–e) indicate significant differences between seats (p < 0.05). See Table 4 for details

Thigh posture varied systematically with hardness (Table 6). At the onset of STS, seat C showed a greater thigh angle than seat E (*p =* 0.002). At PHM, the thigh was more flexed for seat C than for seats D (*p <* 0.001) and E (*p* < 0.001). At PKM, seat C also exhibited a greater thigh angle than seats D (*p =* 0.010) and E (*p* = 0.002).

**Table 6:** Comparison of thigh inclination angle among the three key time points

Seats	Thigh Inclination Angle at the Onset (°)		Thigh Inclination Angle at PHM (°)		Thigh Inclination Angle at PKM (°)	
	Mean ± SD	(95% CI)	Mean ± SD	(95% CI)	Mean ± SD	(95% CI)
A	78.47 ± 4.42	(76.03, 80.92)	72.21 ± 5.29^c^	(69.28, 75.14)	67.73 ± 5.62	(64.62, 70.84)
B	78.80 ± 4.64	(76.23, 81.37)	71.62 ± 5.18	(68.75, 74.48)	67.37 ± 5.84	(64.14, 70.61)
C	78.85 ± 4.69^e^	(76.26, 81.45)	70.91 ± 5.46^a, d,e^	(67.89, 73.93)	68.53 ± 6.98^d, e^	(64.67, 72.39)
D	78.26 ± 4.78	(75.62, 80.91)	68.33 ± 4.84^c^	(65.65, 71.00)	64.40 ± 7.92^c^	(60.01, 68.78)
E	77.46 ± 4.82^c^	(74.79, 80.12)	67.70 ± 5.51^c^	(64.65, 70.75)	63.48 ± 4.92^c^	(60.75, 66.20)

PHM (peak hip moment), PKM (peak knee moment)

(Resilience [%], Hardness [N]) = A(14, 77), B(45, 66), C(53, 76), D(53, 180), E(55, 336)

Superscript letters (a–e) indicate significant differences between seats (p < 0.05). See Table 4 for details

## Discussion

### Effect of seat surface resilience on lower-limb joint loading during STS

This study demonstrated that high-resilience seats significantly reduced the peak hip and knee extensor moments during STS compared with low-resilience seats, which was consistent with our initial hypothesis. In addition, through elastic force, high-resilience cushions were expected to generate greater upward reaction forces on the body. The effects of this tendency were reflected in the timing of seat-off and in the SRF at PHM and PKM, which may have contributed to the observed differences in lower-limb joint moments.

The seat-off phase represents a critical period of instability, during which the base of support shifts from both the buttocks and feet to the feet alone [1]. This transition requires precise control of the COM and coordinated activation of bilateral lower-limb muscles [33], both of which contribute to the increase in joint loading. Generally, lower-limb joint moments tend to increase after seat-off [8, 17, 34]. However, the present study revealed that the timing of seat-off was significantly delayed on high-resilience seats, and in many cases—particularly for seats B and C—occurred after PKM (Fig. 5). This indicates that high-resilience seats tend to delay the timing of seat-off, resulting in PHM and PKM occurring before the base of support is fully transferred to the feet alone and instability increases. Sato et al. reported that, during STS, the time interval from hindfoot to forefoot peak pressure was significantly longer when using a low-resilience foam cushion compared to a seat without a cushion. From this result, they suggested that low-resilience seats prolong the duration between seat-off and postural stabilization [26]. Their findings conceptually align with our observation that, when using low-resilience seats, the proportion of time to reach seat-off relative to the total STS duration was significantly shorter.

Highly resilient materials rapidly return to their original shape after deformation. During STS, the elastic energy returned from the seat surface is released in synchrony with the upward motion of the buttocks, generating an upward reactive force that assists in lifting the body. Consistent with this mechanism, our results showed that greater seat resilience was associated with increased SRF at PHM and PKM. These results indicate that high-resilience seat materials can conform to the motion of the buttocks and are capable of supporting a small portion of the body weight even at the timing when joint loading becomes the greatest.

Taken together, high-resilience seats appear to have the ability to maintain contact with the buttocks for a longer duration and continuously support a small portion of the body weight. Therefore, they may facilitate smoother COM movement during the unstable seat-off phase and ultimately reduce the lower-limb joint loading. These findings suggest that using high-resilience seats may help to reduce hip and knee extensor demands during STS.

### Effect of seat surface hardness on lower-limb joint loading during STS

Our results showed that softer seat surfaces led to a significantly higher peak hip extensor moment during STS, while peak knee extensor moment remained relatively unchanged. In addition, although the difference in peak trunk inclination angle was not statistically significant, the peak horizontal COM velocity was significantly greater. Overall, these findings partially support our second hypothesis, indicating that when rising from a softer seat, a strategy involving greater horizontal momentum generation by the upper body is preferred.

Seat hardness determines the degree of buttock immersion during sitting, thereby influencing the initial posture for STS. In this study, to eliminate the effect of buttock height on the movement, the seat heights were adjusted so that the pelvis marker height was consistent across all conditions. Even with a uniform vertical distance from the pelvis to the floor, softer seats allowed greater buttock immersion, resulting in a more posteriorly tilted thigh posture compared with harder seats. A more posteriorly inclined seat delays the onset of lower-limb muscle activation and prolongs the movement duration [24]; therefore, a softer seat can be considered to provide a disadvantageous initial condition for STS. Consequently, in the soft seat condition, participants likely adopted a strategy involving greater horizontal momentum generation by the upper body to compensate for this disadvantageous posture. Considering that most of the momentum generated by the upper body is transferred to the lower limbs after seat-off [8], this mechanism likely explains the significantly greater peak GRF observed for seat C.

Kuramatsu et al. identified a transition period during which the COM shifts from a forward to an upward trajectory, corresponding to the interval between the peak forward and upward COM velocities [35]. In this transition phase, the hamstrings play a crucial role in controlling the forward movement of the COM and generating hip extensor force to lift it upward [8, 36]. That is, when the forward momentum of the body immediately before seat-off is large, the hip extensor moment increases to decelerate this motion. This mechanism likely explains the larger hip joint moment observed when standing up from the soft seat.

Furthermore, a notable difference in thigh segment angle was observed not only at the onset of the movement but also at the time of PHM. As shown in Table 6, with increasing seat hardness, the thigh inclination angle relative to the vertical axis decreased, indicating that the thighs were positioned in a more forward-leaning posture. This change may have shortened the moment arms of the external forces, thereby reducing the magnitude of the hip joint moment.

Taken together, our results suggest that when rising from softer seats, the increased hip joint moment is primarily attributable to the mechanical disadvantage caused by buttock immersion and the adoption of a compensatory strategy involving greater forward momentum generation. However, previous studies have suggested that this increase cannot be fully explained by postural factors alone. Suriyaamarit and Boonyong reported that although anterior seat inclination reduced the mechanical work required during STS, no significant differences were observed in lower-limb joint moments [15]. Furthermore, Anan et al. demonstrated that thick cushions with greater buttock immersion may reduce the efficiency of energy transfer during seat-off [27]. In the present study, we focused on analyzing segment angles and reaction forces at the timing of the peak joint moments; however, significant differences were limited. Future research should directly examine muscle activation patterns using electromyography to further clarify the mechanisms underlying the increased hip joint moment.

### Practical implications and limitations of the study

Although lift-assist chairs have been proposed to support such movements [25, 37, 38], assistive seat cushions for STS offer practical advantages, including ease of transport and installation, low maintenance requirements, and cost-effectiveness. Therefore, the present results may offer valuable and feasible guidance for both clinical settings and daily caregiving environments.

In this study, healthy young adults were selected as participants instead of elderly individuals, who generally exhibit greater variability in physical and cognitive functions. This design allowed us to clarify the fundamental mechanical effects of seat cushion resilience and hardness on STS under controlled conditions. However, future investigations involving elderly individuals are warranted to extend the applicability of the findings.

When changing seat materials, it was not possible to standardize both seat height and inclination simultaneously. In this study, seat height was prioritized, which may have affected the initial posture and loading conditions. Future studies should carefully consider whether seat height or inclination should be standardized when comparing different seat materials.

While this study did not consider the contribution of the upper limbs, many real-life situations involve occupied hands. Thus, limiting upper limb movement does not compromise the general usefulness of the study. Nonetheless, the involvement of the upper limbs may modify joint loading, joint torque requirements, and postural stability during STS, potentially altering STS and balance control [39, 40]. Thus, future research should also account for the influence of upper limb use during STS.

## Conclusion

The main findings of this study are as follows (1). High-resilience cushions delayed the timing of seat-off and maintained buttock support for a longer duration, resulting in reduced peak hip and knee extensor moments (2). Softer seats, associated with a more posteriorly inclined initial thigh angle, increased horizontal COM velocity and led to greater hip extensor moments, whereas knee joint moments showed little change (3). Within the experimental range, cushions with a resilience of 53% or higher and a 40% compression hardness of at least 180 N effectively reduced lower-limb joint loading. These findings provide fundamental insights into the mechanical influence of seat material properties on STS performance. The results of this study provide practical implications that may contribute to future research on chair design and cushion selection in clinical and caregiving environments.

## Supplementary Information

Below is the link to the electronic supplementary material.

Supplementary Material 1

## Data Availability

The raw data from this study is not publicly available in order to protect individual privacy, in accordance with the policies of the Ethical Review Committee of the Faculty of Science and Technology at Keio University. However, the data may be available from the corresponding author upon reasonable request and with appropriate ethical approval.

## Abbreviations

COM: Center of Mass
GRF: Ground Reaction Force
PHM: Peak Hip Moment
PKM: Peak Knee Moment
RPSI: Right Posterior Superior Iliac Spine
SRF: Seat Reaction Force
STS: Sit-to-Stand
