# Supplementary material for: Effect of seat cushion resilience and hardness on lower-limb loading during sit-to-stand
Source: BMC Biomed Eng. 2026 Jun 17;8:11. doi: 10.1186/s42490-026-00115-w (PMC13277150; doi:10.1186/s42490-026-00115-w)
Supplement: Supplementary file 1 — Supplementary Material 1 [file 42490_2026_115_MOESM1_ESM.docx]

**Appendix**

*Procedure for deriving joint moments*

The joint moments were calculated using the joint center positions obtained using the Vicon Nexus system and the three-dimensional forces obtained using the force plates. In this experiment, the moments applied to the right lower-limb joints were calculated by assuming that the load was equally applied to both sides. The calculation procedure is as follows (Table A1 lists all variables used for the calculations):

1. A rigid-body link model was constructed (Figure A1).


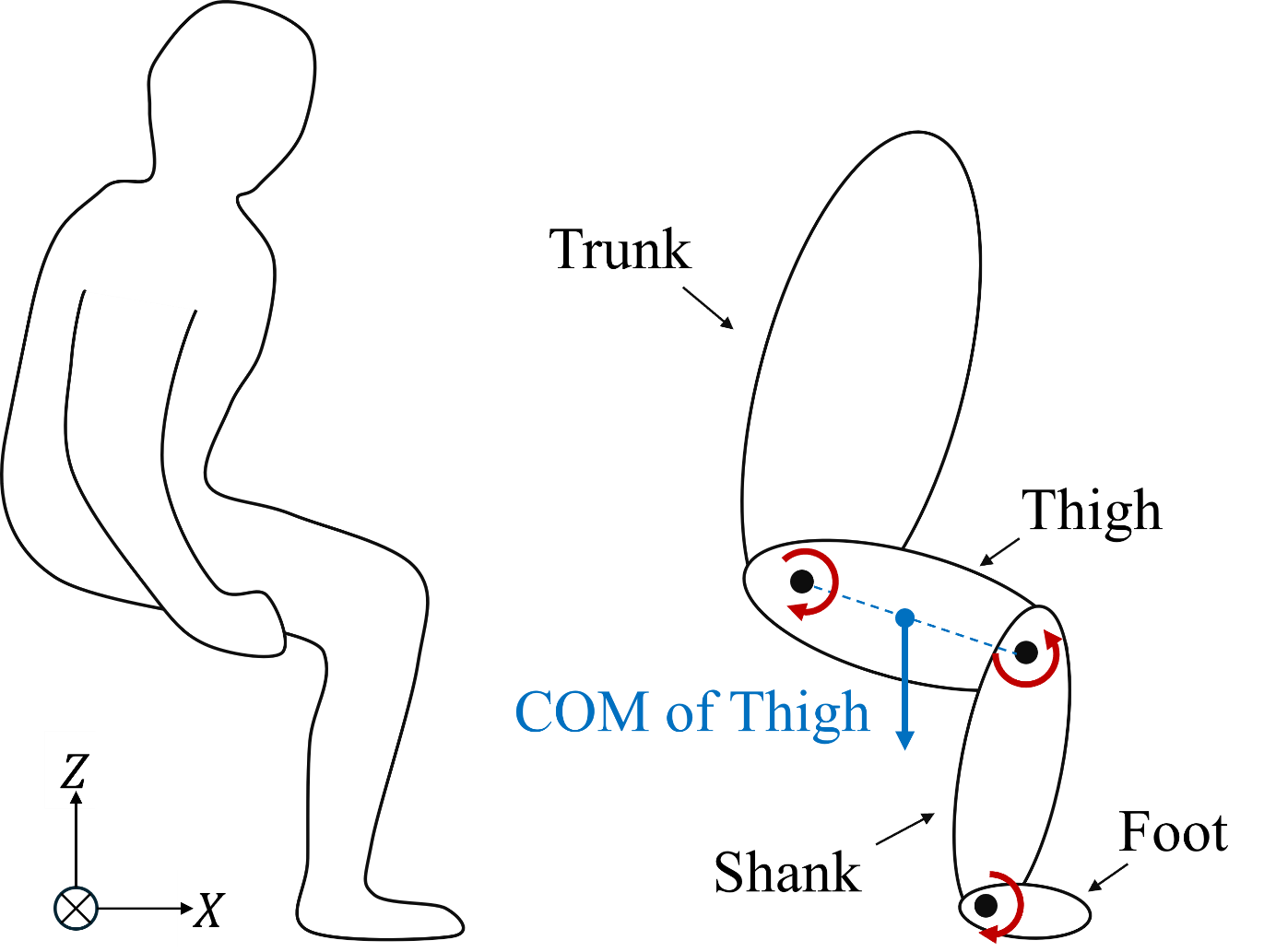


Figure A1 Rigid-body link model.

Four assumptions are made: (a) Each body segment is rigid, and its center of mass is on the axis between joints. (b) Each joint is a uniaxial joint. (c) The trunk, including the head and upper limbs, is a single unit. (d) The motion of each body part occurs only in the sagittal plane.

1. The mass, center-of-mass coordinates, and moment of inertia of each segment were calculated based on a previous study (41).
2. The coordinates of the pressure center of the force plates were calculated using the following equation considering a force plate thickness of 0.047 m:

| $P_{ix} \left[ m \right]=\frac{-M_{\mathrm{iy}}+0.047\cdot F_{\mathrm{ix}}}{F_{\mathrm{iz}}}$ | (1) |
| --- | --- |
| $P_{iy} \left[ m \right]=\frac{M_{\mathrm{ix}}+0.047\cdot F_{\mathrm{iy}}}{F_{\mathrm{iz}}}$ | (2) |

1. Equations of rotational motion around the COM of the foot were established to obtain the ankle plantar flexor moment ($M_{A}$) (Figure A2).

| $R_{AX}=m_{F}\cdot\ddot{x_{F}}-F_{1X}$  $R_{AZ}=m_{F}\cdot(\ddot{z_{F}}+g)-F_{1Z}$  $M_{A}=I_{GF}\cdot\ddot{\theta}_{F}+F_{1Z}\cdot\left( p_{1X}-x_{F} \right)+F_{1X}\cdot\left( z_{F}-p_{1Z} \right)$  $-R_{AZ}\cdot\left( {x_{F}-j}_{AX} \right)-R_{AX}\cdot\left( j_{AZ}-z_{F} \right)$ | (3) |
| --- | --- |


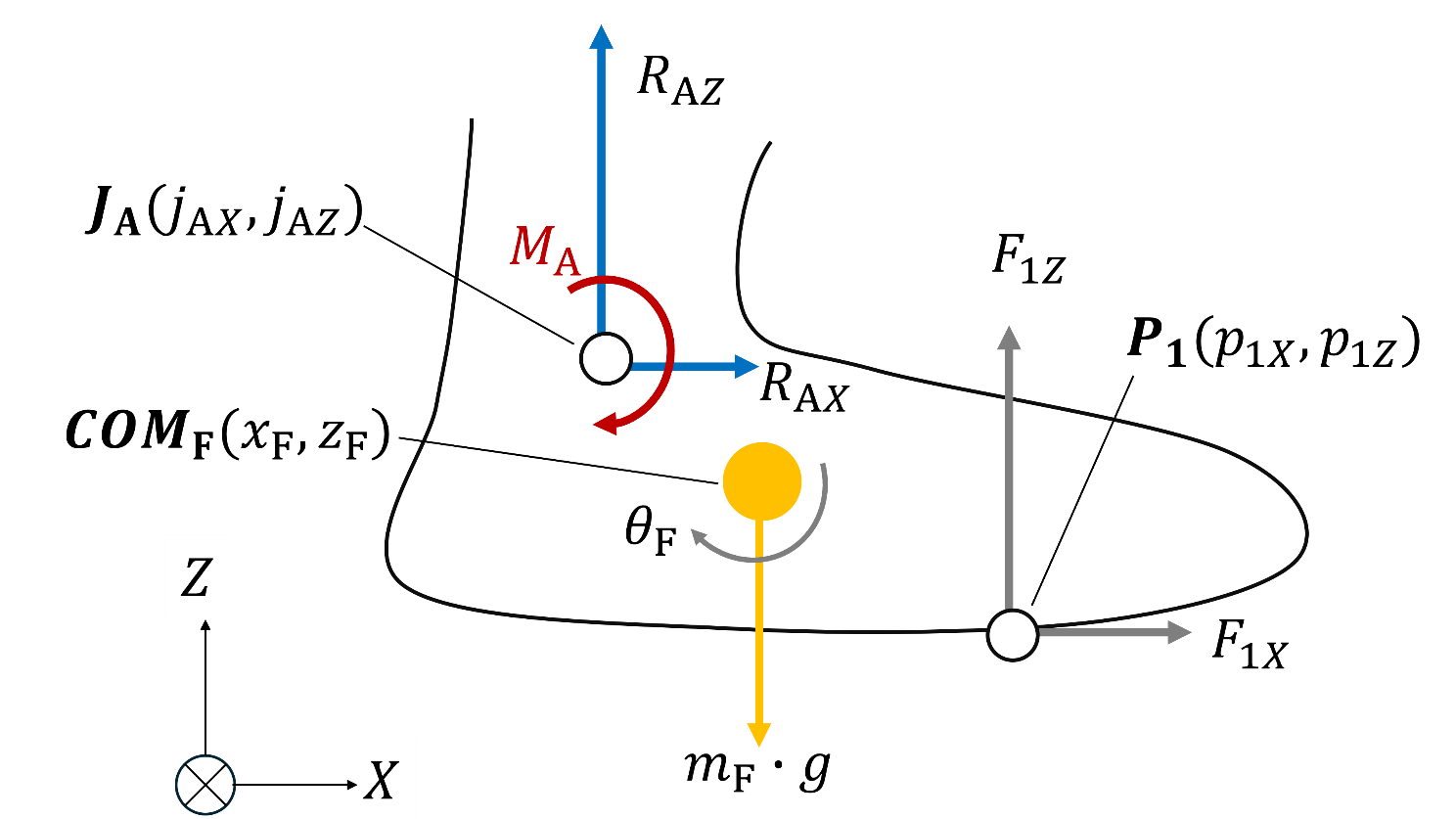


Figure A2 Free-body diagram of the foot segment for calculating the ankle plantar flexor moment.

1. Equations of rotational motion around the COM of the shank were established to obtain the knee extensor moment ($M_{K}$) (Figure A3).

| $R_{KX}=m_{S}\cdot\ddot{x_{S}}+R_{AX}$  $R_{KZ}=m_{S}\cdot\left( \ddot{z_{S}}+g \right)+R_{AZ}$  $M_{K}=I_{\mathrm{GS}}\cdot\ddot{\theta}_{S}-M_{A}+R_{AX}\cdot\left( z_{S}-j_{AZ} \right)-R_{AZ}\cdot\left( x_{S}-j_{AX} \right)$  $+R_{KX}\cdot\left( j_{KZ}-z_{S} \right)-R_{KZ}\cdot\left( j_{KX}-x_{S} \right)$ | (4) |
| --- | --- |


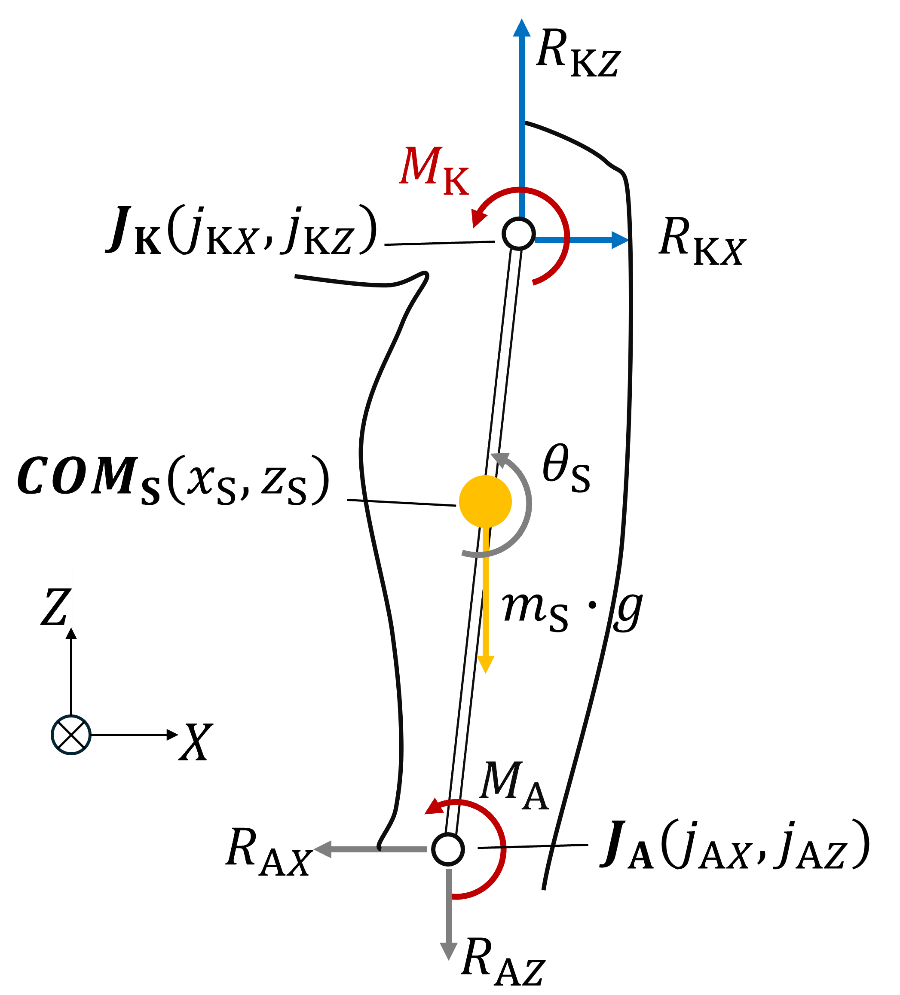


Figure A3 Free-body diagram of the shank segment for calculating the knee extensor moment.

1. Equations of rotational motion around the COM of the thigh were established to obtain the hip extensor moment ($M_{H}$) (Figure A4).

| $R_{HX}=m_{H}\cdot\ddot{x_{H}}+R_{KX}-F_{2X}$  $R_{HZ}=m_{H}\cdot\left( \ddot{z_{H}}+g \right)+R_{KZ}-F_{2Z}$  $M_{H}=I_{\mathrm{GT}}\cdot\ddot{\theta}_{T}-M_{K}-R_{KX}\cdot\left( z_{T}-j_{KZ} \right)-R_{KZ}\cdot\left( j_{KX}-x_{T} \right)$  $-R_{HX}\cdot\left( j_{HZ}-z_{T} \right)-R_{HZ}\cdot\left( x_{T}{-j}_{HX} \right)$  $+F_{2X}\cdot\left( z_{T}-p_{2Z} \right)-F_{2Z}\cdot\left( x_{T}-p_{2X} \right)$ | (5) |
| --- | --- |


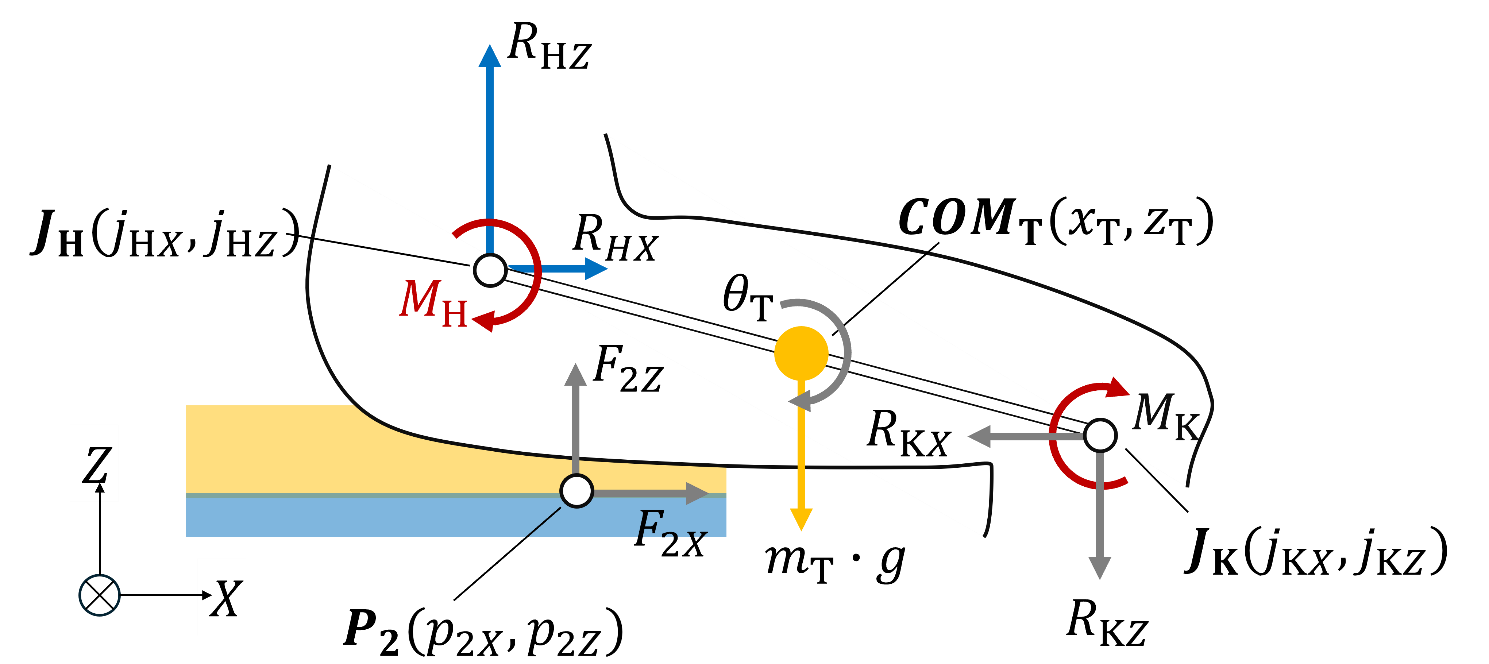


Figure A4 Free-body diagram of the thigh segment for calculating the hip extensor moment.

Table A1 List of variables used to derive joint moments

| Variables | Unit | Variable Description |
| --- | --- | --- |
| $I_{\mathrm{GF}}$ | $kg\cdot m^{2}$ | Inertia moment around the center of gravity of the foot |
| $I_{\mathrm{GS}}$ | $kg\cdot m^{2}$ | Inertia moment around the center of gravity of the shank |
| $I_{\mathrm{GT}}$ | $kg\cdot m^{2}$ | Inertia moment around the center of gravity of the thigh |
| $\boldsymbol{F}_{\boldsymbol{1}}$ | N | Unilateral ground reaction force |
| $\boldsymbol{F}_{\mathbf{2}}$ | N | Unilateral seat reaction force |
| $\boldsymbol{M}_{\boldsymbol{1}}$ | $N\cdot m$ | Reaction moment on the foot |
| $\boldsymbol{M}_{\mathbf{2}}$ | $N\cdot m$ | Reaction moment on the buttocks |
| $m_{F}$ | $\mathrm{kg}$ | Foot mass |
| $m_{S}$ | $\mathrm{kg}$ | Shank mass |
| $m_{T}$ | $\mathrm{kg}$ | Thigh mass |
| $\boldsymbol{R}_{\mathbf{A}}$ | $N$ | Ankle joint reaction force |
| $\boldsymbol{R}_{\mathbf{K}}$ | $N$ | Knee joint reaction force |
| $M_{A}$ | $N\cdot m$ | Ankle plantar flexor moment |
| $M_{K}$ | $N\cdot m$ | Knee extensor moment |
| $M_{H}$ | $N\cdot m$ | Hip extensor moment |
| $\theta_{F}$ | $\mathrm{rad}$ | Foot rotation angle |
| $\theta_{S}$ | $\mathrm{rad}$ | Shank rotation angle |
| $\theta_{T}$ | $\mathrm{rad}$ | Thigh rotation angle |
| $\boldsymbol{COM}_{F}$ | $m$ | Center-of-mass position of the foot |
| $\boldsymbol{COM}_{S}$ | $m$ | Center-of-mass position of the shank |
| $\boldsymbol{COM}_{T}$ | $m$ | Center-of-mass position of the thigh |
| $\boldsymbol{P}_{1}$ | $m$ | Pressure center coordinates of the foot |
| $\boldsymbol{P}_{2}$ | $m$ | Pressure center coordinates of the buttocks |
| $\boldsymbol{J}_{A}$ | $m$ | Ankle joint position coordinates |
| $\boldsymbol{J}_{K}$ | $m$ | Knee joint position coordinates |
| $\boldsymbol{J}_{H}$ | $m$ | Hip joint position coordinates |
